# Abstract of Report on Etiology, Physiology, Prophylaxis, Hygiene, and Electricity

**Published:** 1901-09-15

**Authors:** J. D. Patterson

**Affiliations:** Kansas City, Mo.


### ABSTRACT OF REPORT ON ETIOLOGY, PHYSIOLOGY, PROPHYLAXIS, HYGIENE, AND ELECTRICITY.

BY J. D. PATTERSON, D.D.S., KANSAS CITY, MO.

---

Dr. Patterson's report was a resume of the year's progress in Etiology, Physiology, Prophylaxis, Hygiene and Electricity.

Regarding the subject of dental caries, he cited the discovery of "gelatinous plaques" by Black and Williams and recently promulgated knowledge about caries brought forward at the recent International Congress at Paris. He spoke of Dr. Michael's report on the saliva, who claimed that "the saliva, like all recrementitious secretions, is liable to physiological oscillation in constant relation to the humors of the body.'' Connecting this study with caries of the teeth, these conclusions with others are laid:

1. ''The saliva contains definite chemical principles which arrest or retard the progress of dental caries (sulfocyanid of ammonia).''

2. ''The alkaline sulfocyanids arrest the formation of putrefactive fermentation.''

3. ''Chemically, dental caries is a disease of demineralization, due to the presence of an excess of acid principles in the saliva having a greater chemical affinity for the constituents of the tooth.''

4. ''The chemical affinity of potassium and sodium is greater than that of the earthy alkaline bases in combination with acid salts.''

5. ''Lactic acid possesses a greater affinity for calcium than does carbonic acid.''

6. ''Active dental caries characterizes the hyperacid diathesis.''

7. ''Diathetic dental caries (gout and diabetes) results from lactic acid fermentations.''

Regarding hygiene, Dr. J. D. Logan, of Edinburgh, says: ''Having discussed the matter of caries in relation to health and the condition of the oral cavity generally in relation to the dental organs, we may ask wherein lies the cause of this appalling destruction of teeth. Verily the answer is not difficult to find; it must be entirely one of hygiene.

As a prophylactic mouth-wash, Dr. G. V. I. Brown has advocated peroxid of hydrogen as superior to solutions of carbolic acid and bichlorid of mercury, which, when held in the mouth, destroy the superficial germs, but not those deeper located, while peroxid of hydrogen oxidizes the organic deposits about the teeth, loosens up the secretions about the gums, and sets free germs that were at first inaccessible to the action of carbolic acid or bichlorid of mercury. After the germs have been thus set free, any of the above solutions will bring about a desirable state of asepsis.

The essayist spoke about the inspection of the teeth of school children in Europe and advocated such a movement in America.

Regarding the etiology of sensitive dentin, Dr. Gysi, of Zurich, holds the following theory: "Substances rich in water are incompressible. The dentin is traversed by a great number of canaliculi which are filled with a substance very rich in water (about 80 percent), and which, like water, is incompressible. When pressure is exercised in a carious cavity with an instrument, the pressure is directly transmitted through the semi-liquid contents to the odontoblasts, which are the center of the true sensitiveness of the dentin."

The subject of pyorrhea was discussed, and the various theories, already familiarly known, were reiterated.

DISCUSSION.

Dr. C. N. Pierce, of Philadelphia: I am not satisfied that micro-organisms are always responsible for dental caries. I am of the opinion that more frequently defective enamel and other tooth structure have much to do with it. And general systemic conditions, which produce abnormal buccal secretions and functions, should not be overlooked. Caries is most active during the years from seven to twenty, or at the time when the alkaline material is being selected for the building of the hard tissues, leaving the normal fluids acid in character. This condition would favor acid buccal secretions and be likely to bring on erosion.

I would classify suppuration of the gums into two classes, as true and false pyorrhea. True pyorrhea is systemic in character, because it is due to a condition of perverted nutrition, or rather of perverted excretion. It may, however, be developed locally by local irritation, such as injury to the gums by scaling the teeth or other injuries which are capable of setting up infectious conditions. The false pyorrhea is a condition which results from local infection only, without systemic complications. This kind is amenable to local treatment.

Dr. W. C. Barrett, of Buffalo, N. Y.: The report reminds me of a beautiful piece of patchwork. The threads, however, do not always hold its pieces together in proper form. None of the points noticed have been adequately presented. Miller has fully demonstrated that some micro-organisms which do not usually generate acid are capable of producing dental caries. The environment of the teeth has much to do with the rapidity and extent of this disease. It is a reproach to dentists that we can not clearly define the cause and nature of pyorrhea alveolaris. Some scientific investigator will solve this problem and immortalize himself in doing so. About all that we certainly know is that it is a peridental disease.

Dr. E. S. Talbot, of Chicago, expressed surprise that this disease should ever be referred to as pyorrhea alveolaris, because it often exists without pus. There always is inflammation, superficial or deep-seated, involving the interstitial structures. "Interstitial gingivitis" is a more fitting name. The soft tissues are always involved, and generally the hard structures are sooner or later implicated. There is no such differentiation as "true and false" to be made in this disease.

Dogs, experimentally treated with mercury, will show indications of periosteal inflammation in various stages. Impaired eliminatory function will produce localized pericementitis. The hot weather in the Philippines has shown its influence in producing "interstitial gingivitis" or pyorrhea. In the cold mountains, at high altitudes, we find the people suffer from interstitial gingivitis; because of impaired skin function there accumulate toxic influences in the blood tissues, and from such affected blood we have auto-intoxication. We have the same results as a secondary effect of asthma. In fact, we may oftentimes diagnose liver or kidney diseases from the smell of the breath or the condition of the gums. Such diseases produce degenerative changes in the arterial system, usually localized, and nowhere possibly more often than in the gums. These inflammatory conditions are of a low and chronic grade, and may not change to the suppurative condition for years after they become affected. The deposits on the teeth are only incidental and in no sense causative.

---

Dr. C. L. Hungerford, of Kansas City, Mo., read a paper on the general subject of "The Etiology of Disease." The paper was metaphysical in character and exceedingly difficult to report. In fact, it was so little understood that many present supposed it was advocating Christian Science, and two or three devotees of this sect discussed it from that standpoint very fervently and at considerable length. As nearly as we could comprehend the paper, it was an attempt to theorize as to the transmigration of matter and the influences made upon matter in its various habitats; and again, the influence of matter contaminated by its various habitats upon the various organisms into which it enters and helps to form in its cycle of existence. We have always thought of matter, in its atomic form at least, as pure, if not sterile, consequently not capable of contamination because of its accidental surroundings. It may be possible for molecular matter to suffer contamination, but this subject is too much for us. The following sentences will perhaps give our readers some idea of the thought of the essayist:

"Habit is contrary to the law of evolution. Habit continued produces a physiological function. Pain is a symptom of disease and indicates interference with function. Matter always existed, and affinity of matter accounts for cell, tissue and organ. How does the cell acquire a habit? Good health or disease are equally catching. The object of life is to attain perfection; good and evil are only conditions. Pain is the outcome of evil. Mental attitude has much to do with exciting or depressing normal function. The forces of the universe tend to establish an equilibrium, and forces of evil import tend to disease or disturbance. If a man has no mental niche into which a fact may be shot, so much worse for the fact."

### DISCUSSION.

Dr. Jas. Truman, of Philadelphia: I can't talk on a metaphysical subject on three minutes' notice, and don't know anything about Christian Science. Have always believed that disease may be aggravated or influenced by mental activities. The mental attitude of a patient has great influence, also, in enabling us to perform operations on sensitive structures, but I am not prepared to intelligently discuss this phenomena at this time.

Dr. Albrecht, of Chicago: I was cured of eczema of six years standing by taking the absent treatment for six weeks, my physician living in Indianapolis whilst I was in Chicago. I have treated my own patients for attacks of headache, spasms of coughing and severe colds, in fifteen minutes' time, so that I was able to make dental operations for them with comparative comfort, and consequently feel that applied metaphysics is a great blessing.

Dr. J. W. Cormany, of Chicago: A thought has power, and will go where sent and do what it is sent to do. I had a brother ill in a Cincinnati hospital with an infectious disease, and in spite of the fact that his physicians gave no hopes of his recovery I applied the absent treatment from my home in Chicago and saved his life.

Dr. J. N. Crouse, of Chicago: There is no question but that a distracted mentality will influence abnormally the habitual body functions, producing indigestion, etc. I have several patients, one in particular, for whom I used to operate with the greatest difficulty, but upon whom now, under what she terms "right living," I am able to make the most severe operations without unusual suffering on the part of the patient.

Dr. Hungerford, in closing the discussion, said that he had been greatly misinterpreted by most of the speakers, and that, whatever else may have been expressed or implied in his paper, Christian Science was not. In fact, the ideas of his paper made Christian Science an impossibility.

